# Focus perception in Japanese: Effects of lexical accent and focus location

**DOI:** 10.1371/journal.pone.0274176

**Published:** 2022-09-22

**Authors:** Albert Lee, Faith Chiu, Yi Xu

**Affiliations:** 1 Department of Linguistics and Modern Language Studies, The Education University of Hong Kong, Tai Po, Hong Kong SAR; 2 Department of Language and Linguistics, University of Essex, Colchester, United Kingdom; 3 Speech, Hearing & Phonetic Sciences, Division of Psychology & Lang Sciences, University College London, London, United Kingdom; Tohoku University, JAPAN

## Abstract

This study explored the contexts in which native Japanese listeners have difficulty identifying prosodic focus. Using a 4AFC identification task, we compared native Japanese listeners’ focus identification accuracy in different lexical accent × focus location conditions using resynthesised speech stimuli, which varied only in fundamental frequency. Experiment 1 compared the identification accuracy in lexical accent × focus location conditions using both natural and resynthesised stimuli. The results showed that focus identification rates were similar with the two stimulus types, thus establishing the reliability of the resynthesised stimuli. Experiment 2 explored these conditions further using only resynthesised stimuli. Narrow foci bearing the lexical pitch accent were always more correctly identified than unaccented ones, whereas the identification rate for final focus was the lowest among all focus locations. From these results, we argue that the difficulty of focus perception in Japanese is attributed to (i) the blocking of PFC by unaccented words, and (ii) similarity in F0 contours between lexical pitch accent and narrow focus, including in particular the similarity between downstep and PFC. Focus perception is therefore contingent on other concurrent communicative functions which may sometimes take precedence in a +PFC language.

## Introduction

Focus is a communicative function for directing the listener’s attention to information that the speaker believes is especially important [1, 2]. For those languages which employ fundamental frequency (F0) as a cue to mark focus, a natural question arises as to whether there is a conflict or competition between focus and other communicative functions (e.g., lexical tone / accent) which are also expressed mainly by F0. A further question is whether the effectiveness of F0 as a prominent cue in conveying focus varies across different focus locations. This question stems from the fact that, in many languages, focus is conveyed by multiple markers (e.g. prosodic, syntactic, morphological), each of which can employ one or multiple phonetic alterations of various cues such as duration, intensity, and F0 [e.g. 3 on Finnish]. However, a good understanding of the interaction of these surface cues is only possible after investigating the role of each of these particular phonetic cues when independently manipulated. The questions about how focus may interact with other functions which too control F0 could be answered by a perception task in which F0 is manipulated while other aspects of speech are held constant. This can be achieved through a speech resynthesis tool called PENTAtrainer that we have developed [4].

### Focus markers in Japanese

To mark narrow focus in Japanese, native speakers can use a combination of syntactic [see 5 for a review], morphological (i.e. using the focus particles *dake* ‘only’ or *mo* ‘too’), and prosodic strategies. Prosodic cues to narrow focus include, acoustically, on-focus F0 range expansion and post-focus F0 range compression [PFC henceforth, 6–9] alongside the modification of non-F0 cues such as duration and formant frequency [10]. These prosodic cues were identified in production experiments when participants were disallowed from using any of other aforementioned non-prosodic focus-marking strategies.

On the other hand, how native listeners perceive narrow focus is not as well understood. While it is easy to instruct speakers to produce focus, it is next to impossible to get participants to produce it with only one of the possible prosodic cues. It is well known that beside F0, focus also affects duration [10, 11], voice quality [12] and formant frequency [10], all of which could also serve as secondary cues in focus perception. Because F0 is involved in a wide range of communicative functions (e.g., focus, emotion, sentence type), and a given prosodic pattern (e.g., a raised F0 peak) could be associated with a number of different meanings, how well native listeners perceive focus with only F0 cues available warrants investigation. This is interesting because when all the secondary cues are held constant, it is possible that F0 patterns associated with focus alone would not be very effective.

There are very few studies that have systematically investigated focus identification in Japanese, with exceptions such as [13]. To verify the naturalness of their production data from three speakers, they had 20 native listeners participate in a 6AFC identification task in which narrow focus was one option; the other options were ‘admiration’, ‘suspicion’, ‘disappointment’, ‘indifference’, and ‘neutral’. Their results showed that correct identification of focus varied greatly across the three speakers, ranging from 23% to 77% (ibid.). As all the three speakers were native experienced teachers of Japanese language, it is surprising that narrow focus in their production would be so poorly identified by the native listeners. Also, the huge discrepancy in focus identification between the productions of different speakers means that rather different acoustic cues may be employed. It is thus necessary to consider using resynthesised speech research on focus perception, so that it is possible to control one acoustic parameter while others are held constant.

### Effect of lexical prosody

F0 is an acoustic dimension that has been shown to be involved in cuing multiple communicative functions [see, for example, introduction by 14]. For languages that use F0 to mark both lexical prosody (e.g., tone or lexical pitch accent) and focus, it is an intriguing question how listeners simultaneously decode the multiple pieces of information from the F0 signal.

For example, the role of lexical prosody in focus perception has been reported for a tone language like Mandarin. Mandarin has four lexical tones (High, Rising, Low, and Falling), each differing from the others in terms of F0 movement direction (alongside other cues). Theoretically, these four ‘full’ tones are considered equal in prominence, as opposed to the Neutral Tone which is produced with weaker articulatory effort [15]. In a perception study, [16] showed that native Mandarin listeners identified focus much less accurately when it was on the Low tone than on the other tones. They attributed this discrepancy to the fact that the Low tone in Mandarin has a smaller capacity for F0 range expansion and a relatively weaker intensity. This is interesting because unlike culminative word prosody systems (e.g., stressed vs. unstressed syllables in English and accented vs. unaccented mora in Japanese), the ‘full’ tones in Mandarin are presumably equal in prominence. If a given tone category can stand out as being more poorly identified for focus in a language where every syllable is specified for tone, it would be interesting to ask how large the discrepancy would be in a culminative word prosody system where one tone category is naturally more prominent than the other. The lexical pitch accent system of Japanese offers a perfect test case for this question.

In Japanese, a word can be either *lexically* accented (accented henceforth) or unaccented; for an accented word, the pitch accent could fall on any syllable. The lexical pitch accent (pitch accent henceforth) in Japanese, or its lack thereof, serves to mark lexical contrasts. Acoustically, it bears a high falling F0 pattern [17]. For example, in *ha’shi* ‘chopsticks’ the pitch accent falls on the first syllable, and pitch shows a high-low pattern; in contrast, *hashi* ‘edge’, which is unaccented, is phonologically assigned a LH pitch pattern. Unlike lexical tones, of which all members are deemed equal in prominence within a language [except, for example, the Neutral Tone in Mandarin which is ‘weaker’ than other tones, 18], an accented mora in Japanese stands out among unaccented ones (which bear a relatively level F0 pattern, ‘H-’ in J-ToBI, the prevailing annotation convention for Japanese prosody [17]). Acoustically, the pitch accent differs from unaccented words with a higher F0 peak followed by a steep fall [8, 19]. The F0 movement of the pitch accent allows more room for F0 range and intensity variation compared with unaccented words, much like the Mandarin tones compared with the Low tone in [16].

Between accented and unaccented words, there are both theoretical and phonetic reasons to consider the former being more prominent. Within J-ToBI, unaccented words are marked as bearing the default melody of a prosodic word (%L H-), whereas accented words are additionally marked by H*+L, i.e. %L H- H*+L. Acoustically, the H* tone is perceptually salient with both higher F0 scaling and stable alignment [20]. As such, it is reasonable to assume that in a neutral (i.e. broad) focus utterance where an accented word is surrounded by unaccented words (i.e. unaccented-accented-unaccented, henceforth UAU), the accented word would stand out and be more prone to being misperceived as bearing a narrow focus. It is thus likely that a UAU utterance under neutral focus would be the most easily confused with medial focus and yield the lowest focus identification accuracy, among all the accent conditions.

### Effect of focus location

Another likely source of difficulty in focus perception is focus location. In the literature on Japanese focus production, various narrow focus conditions have been either reported or predicted to be confusable with neutral focus:

#### Initial focus

In their review of prominence marking in Japanese prosody, [21] argued that initial focus and neutral focus might be ambiguous because ‘there has to be at least one IP-initial rise at the beginning of every well-formed utterance (in Japanese). That is, when there is no narrower focus prompting an IP break and reset later on, the rise from the utterance initial [%L] makes the next immediate [H] target (whether a phrasal [H–] or the [H] of a [H*+L]) the highest (most prominent) peak in the utterance’ [21] (in J-ToBI, the IP (Intonational Phrase) ‘is the prosodic domain within which pitch range is specified…’ [17]).

**Fig 1:**
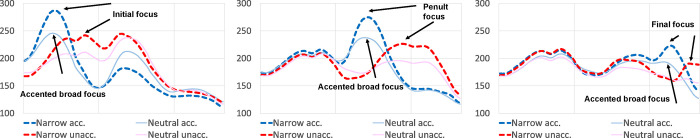
Averaged F0 contours of initial / penultimate / final narrow vs. neutral × accented vs. unaccented focus [data from 9].

The left panel in Fig 1 [data adapted from 9] illustrates this scenario: when compared with neutral focus (Fig 1, solid blue line), the initial narrow focus contour (dashed blue) shows clear evidence of on-focus raising at the beginning of the utterance and PFC in the middle and ending parts of the utterance. However, when inspected individually, both of these two contours are characterised by a high utterance-initial peak which could mark either narrow focus or pitch accent, and by a lowered peak in the middle of the utterance which could be either caused by PFC or by downstep (‘downstep’ refers to the lowering effect of a low tone on a following high tone, such that a new, lower, ceiling is set on all subsequent high tones in a given domain [22]). In other words, where F0 is not reset utterance-medially by a later narrow focus, the highest peak will always be on the first word of the utterance. Meanwhile, when narrow focus is utterance-initial, on-focus expansion will raise the first peak, but will not change the fact that it is the highest in the first place. Thus this case likely leads to ambiguity for the listener as they cannot be sure if the initial peak is raised by focus or is intrinsically high due to normal realisation of an early pitch accent.

#### Penultimate focus

Unlike initial focus, there is evidence that for Japanese (and other subject-object-verb, or SOV, languages) there is no PFC after a penultimate focus, leaving on-focus raising as the only cue available [6, 7, 9]. This is considered to be due to the ‘focus projection’ principle. Focus projection predicts that placing prosodic focus on the object noun phrase (NP) leads to two possible interpretations: narrow focus on the NP and broader focus on the verb phrase (VP). It follows that for an SVO language, like English, final focus and broad focus on the VP would be ambiguous [23–25] whereas for a SOV language like Japanese broad focus on the VP would be indistinguishable from narrow focus on the object NP, i.e. penultimate focus [6, 7, 26] (see also the middle panel of Fig 1). The same has also been observed in Turkish, another SOV language [27]. The distinction between the two focus conditions when produced in laboratory speech is marked by on-focus F0 raising, and PFC appears to be absent (overlapping blue contours towards the end). Thus, compared to initial focus, listeners have one less cue to rely on in penultimate focus. Because of this, focus perception may be more difficult in this position. See [5] for a review of relevant literature on the syntax-prosody interface in Japanese.

#### Final focus

Across languages it has been shown that final focus is prosodically expressed much less effectively than an earlier focus [11, 28–31]. In English, for example, an utterance-final word bearing narrow focus is produced with less relative emphasis [30]. For SVO languages, part of the reason would be complications due to focus projection, as discussed above. Meanwhile, [32] suggested that this could be the result of the conflicting needs to encode both sentence type (questions vs. statements) and focus in the sentence-final word. As Japanese also marks questions with an utterance-final F0 pattern (boundary tone) [8], an overladen utterance-final word would have reduced space for F0 modification for focus, possibly leading to ambiguity. If acoustic cues in production are ambiguous in the first place, it is reasonable to expect that listeners are also easily confused in perception. Fig 1 (right panel) shows that although there is clear evidence of on-focus raising that separates narrow from neutral focus, the pre-focus portions of the F0 contours largely overlap. How sensitive listeners are to the F0 difference in the final word alone would hence determine their ability in identifying narrow final focus.

Given the above-listed issues, the first goal of this study is to find out how Japanese listeners’ perception of narrow focus can be affected by pitch accent. Secondly, we want to determine if focus location has a clear impact on focus perception in Japanese, and if so, whether it is initial, medial or final focus that can be most affected. Finally, we are interested in how well listeners can identify focus when F0 is the only cue. These questions will be answered by a series of perception experiments.

## Experiment 1: Pilot study

Experiment 1 explores the possibility of using resynthesised stimuli for focus perception experiments. This is because resynthesised speech is better controlled and free from cross-repetition variations unlike naturally produced stimuli, and as such, it would be theoretically better to test focus identification using resynthesised stimuli. However, it is unclear if listeners would perceive focus on resynthesised stimuli differently from natural ones. We thus compared listeners’ focus perception across natural and resynthesised stimuli in this experiment.

Our goal is to test the effects of focus location and accent condition on focus identification using resynthesised stimuli, which are better controlled and free from cross-repetition variations that are common in natural stimuli. To achieve this goal, it is necessary to first establish that the resynthesised and natural stimuli are not significantly different in focus perception. In this pilot experiment, we compared how participants respectively performed with the two types of stimuli.

### Method

#### Natural stimuli

Both naturally produced and resynthesised stimuli were used in this experiment. The target sentences, adapted from [9], were designed to elicit quasi-minimal contrasts in F0 patterns in a production experiment (see Table 1). In choosing these target sentences, several factors were taken into consideration: (i) they should be as similar to one another as possible in terms of segmental contents (e.g. same vowel height, consonant manner) so as to directly test the effect of F0; for the same reason, (ii) they should not contain any non-F0 cues to focus such as the marker *dake* ‘only’ that modifies noun phrases or -*noda* attached to final verb phrases, and (iii) they should be identical in length, which can affect F0 range due to soft pre-planning [33]. While yielding semantically less natural sentences, our design ensured strict experimental control that allowed us to assess the effects of focus on F0 contours as well as the effects of F0 variations as cues for focus perception. As will also be explained in the General Discussion section, these target sentences have elicited responses in line with comparable studies in the focus prosody literature.

**Table 1:** Stimuli used in the present study [adapted from 9]. For easy illustration, here the accusative case marker–*o* (which collocates with *mita* ‘saw’) and the dative case marker–*ni* (which collocates with *nita* ‘resembled) are presented as though belonging to Word III; syntactically they are part of Word II.

		Word I		Word II		Word III
Short	Accented	Me’i-ga May-NOM	×	mo’mo thigh	×	-o mi’ta -ACC saw.
	Unaccented	Mei-ga Niece-NOM		momo peach		-ni nita -DAT resembled.
Long	Accented	Mu’umin-ga Moomin-NOM	×	bu’dou martial arts	×	-o mi’ta -ACC saw.
	Unaccented	Noumin-ga Farmer-NOM		budou grapes		-ni nita -DAT resembled.

In the original corpus (N = 6,400), each utterance was either eight (short) or 11 (long) morae in length so as to compare the course of F0 movement under different utterance length conditions. For each word location, an initially accented (e.g., HLL) word and an unaccented (e.g. LHH) word were compared, yielding eight possible combinations of pitch accent condition (two accent conditions × 3 word locations). There were four possible focus conditions for each target sentence, namely initial, medial (i.e., penultimate), final, and neutral (i.e., broad). The sentence types were yes/no questions vs. statements. Narrow focus was elicited by having the speaker produce a given sentence first as a question then as a (corrective) statement in pair.

The natural stimuli used in this study were produced by a 33-year-old female native Japanese speaker from Greater Tokyo (born in Tokyo, grew up in Kanagawa) who worked as a professional voice-over actress in London. Recording took place in a sound-attenuated booth in University College London, using a RØDE NT1-A microphone. The sampling rate was 44,100 Hz. The speaker was seated in front of a computer screen, on which stimuli were displayed one by one in random order. From Table 1, one utterance of each of the long target sentences (N = 64, i.e., eight accent conditions × four focus conditions × two sentence types) was randomly chosen. Short utterances were not included in order to reduce the total number of trials. For details of the acoustic analysis of the original corpus, please refer to Fig 1 for averaged F0 contours in some of the accent conditions and [9] for full details.

The natural stimuli (N = 64) were analysed using ProsodyPro [34]. Speech data were first segmented into morae (where a light syllable is one, e.g., *ta* ‘field’ and a heavy syllable is two, e.g., *tan* ‘phlegm’). Vocal pulses detected by Praat were manually checked and rectified. Because of the controlled nature of the experimental setting, we were able to yield consistently produced utterances with highly comparable F0 patterns and good accuracy. The speaker produced on-focus raising of F0 on the whole word, rather than on the following case marker only [see discussion in 21]. In general, unless there is a later narrow focus (i.e., medial or final), each utterance constitutes one Major Phrase with no evidence of subsequent pitch reset. Paired samples t-tests showed that the word produced under narrow focus was often significantly higher in mean F0 than its neutral focus counterpart. For initial focus, it was 19.69 Hz (SD = 17.99) higher, *t*(15) = 4.38, *p* = .001 (two-tailed); for medial focus, it was 17.05 Hz (SD = 26.13) higher, *t*(15) = 2.61, *p* = .020; for final focus, the difference was non-significant.

#### Resynthesised stimuli

The resynthesised stimuli were generated using PENTAtrainer [4]. PENTAtrainer is a semi-automatic software package for analysis and synthesis of speech melody based on an articulatory-functional model [35]. The following steps were taken during stimulus generation: (i) data preparation, (ii) functional labelling, (iii) model training, and (iv) F0 synthesis, as will be described in more detail below.

Based on the Parallel Encoding and Target Approximation (PENTA) Model [35], PENTAtrainer extracts function-specific underlying pitch targets (target height, target slope, and target strength) by means of analysis-by-synthesis [4]. The pitch targets are articulatory goals that are approached within user-defined tone-bearing unit, which is always the syllable in our own practice e.g. [36]. The articulatory strength of a target specifies how fast the target is approached. Users annotate communicative functions in tiers in the form of Praat TextGrid interval labels. The programme then automatically learns the pitch targets through analysis-by-synthesis controlled by simulated annealing, a stochastic machine learning algorithm [37]. The learned pitch targets each correspond to a unique combination of multiple communicative functions (e.g., H + Question + pre-focus + Left Edge of Sentence), which can be used to generate F0 contours that can be directly compared with natural utterances [4]. The accuracy of synthesis (measured in terms of Pearson’s *r* and root-mean-square error) of PENTAtrainer has been reported to be outstanding [e.g. 36, 38], rendering it particularly suitable for our purpose–to test focus identification using accurately resynthesised, natural-sounding stimuli. In fact, [39] reported that PENTAtrainer could resynthesise the original corpus on which the present study was based as accurately as Pearson’s *r* > .90 (i.e. comparing F0 data of natural utterances and corresponding resynthesised utterances). This high level of synthesis accuracy led us to choose PENTAtrainer to generate the stimuli used in this study.

Firstly, to obtain accurate F0 trajectories, vocal pulses were manually checked and rectified with ProsodyPro for all the natural utterances produced by the aforementioned speaker (N = 640, i.e., eight accent conditions × four focus conditions × two sentence types × two lengths × five occurrences). This step was necessary because F0 estimation can be imprecise, particularly during creakiness. The recordings were then segmented by the mora in Textgrid files. In this case, heavy syllables (i.e. CVV and CVn) were labeled as two intervals equal in duration by inserting an interval boundary in the middle of the syllable.

Then, the resultant data were labelled in terms of communicative functions [35], each in a separate tier in the TextGrid. In this approach, the labels of speech recordings are blind to actual F0 contours, unlike the more common practice of annotation based on phonetic realisation [17]. It is based on the assumption that communicative functions, such as ‘tone’, ‘focus’, ‘sentence type’, ‘emotion’, are the underlying categories that generates surface F0 contours through an articulatory process that can be simulated by the target approximation model [40] as the core of the PENTA model. In PENTAtrainer, communicative functions as well as their internal components are considered as hypothetical whose phonetic values are learned from natural speech data through computational optimisation. Researchers can continually refine their labelling schemes to find out the optimal combination of communicative functions for a given corpus.

**Fig 2:**
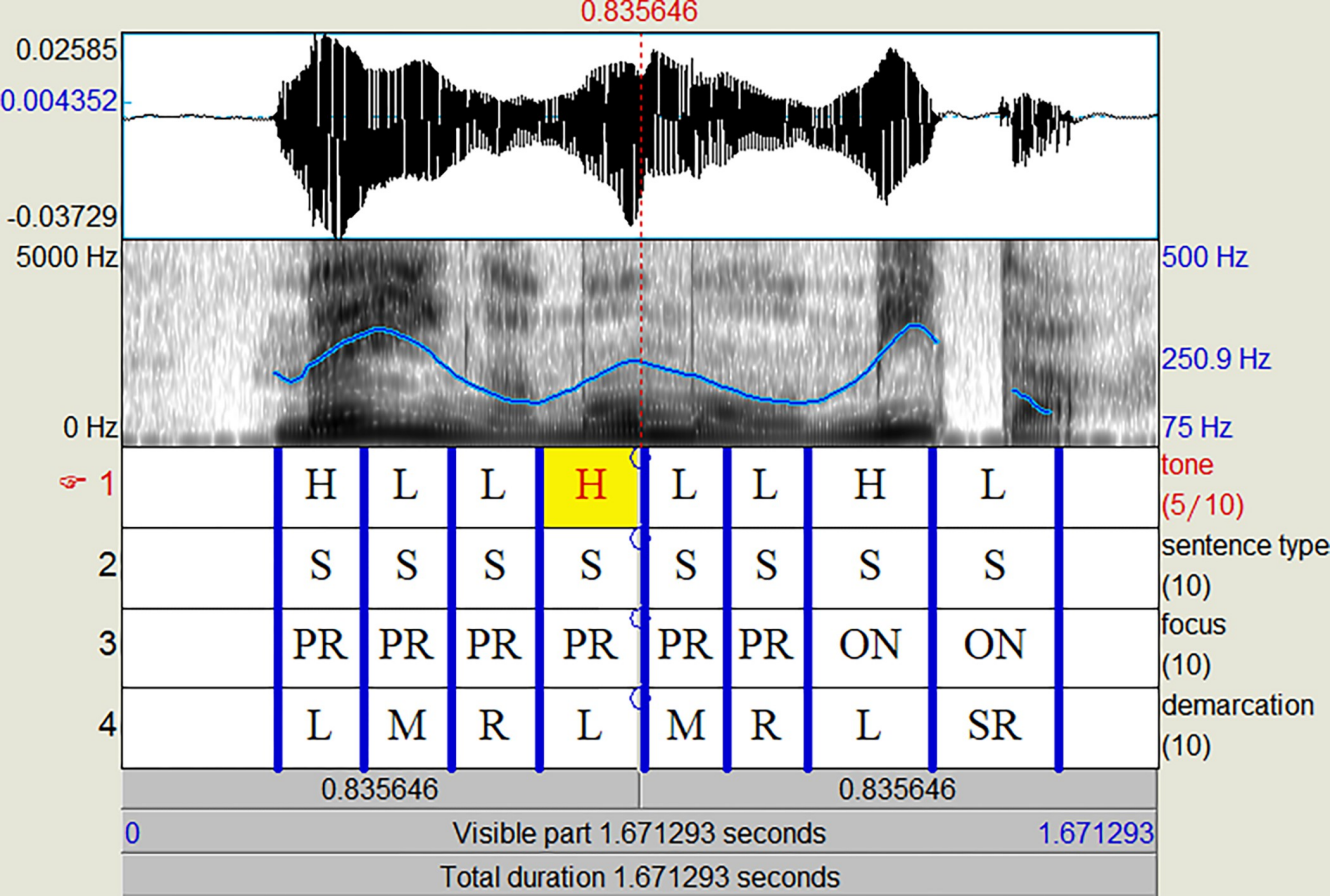
Example of functional labelling using PENTAtrainer.

Fig 2 illustrates how functional labeling was performed in this corpus. For the present corpus, four communicative functions were labeled, namely Tone [with the labels ‘H’ and ‘L’ for accented words and ‘M’ for unaccented words, following 35], Sentence Type (“Question” and “Statement”), Focus [“pre-focus”, “on-focus”, and “post-focus”, following 41, and “neutral”] and Demarcation (“Left Edge of Sentence”, “Right Edge of Sentence”, “Left Edge of Word”, “Right Edge of Word”, and “Medial”). As in [36], “H” and “L” in the Tone tier are associated with the pitch accent, where “H” marks the accented mora, and “L” represents the tone following “H”. Meanwhile, “M” indicates the tones in an unaccented word. Note that sentence length was not included in the model as the effect of length on F0 realisation is considered to be predictable and determined by the Target Approximation mechanism [40]. The order of the tiers in Fig 2 is irrelevant as communicative functions are considered to be parallel to each other [35] and are implemented accordingly in PENTAtrainer [4]. See [39] for more details regarding resynthesis procedures and [36] for a comparable study using a different corpus (single-word Japanese utterances).

In the next step, PENTAtrainer extracted the pitch target parameters (in terms of target height, target slope, and articulatory strength) for each combination of the four communicative functions through analysis by synthesis. This means that from our training corpus (N = 640), 72 sets of parameters were extracted. With these parameters, PENTAtrainer then generated F0 contours which were imposed onto the segmental materials of the natural utterance to form the resynthesised stimuli.

**Fig 3:**
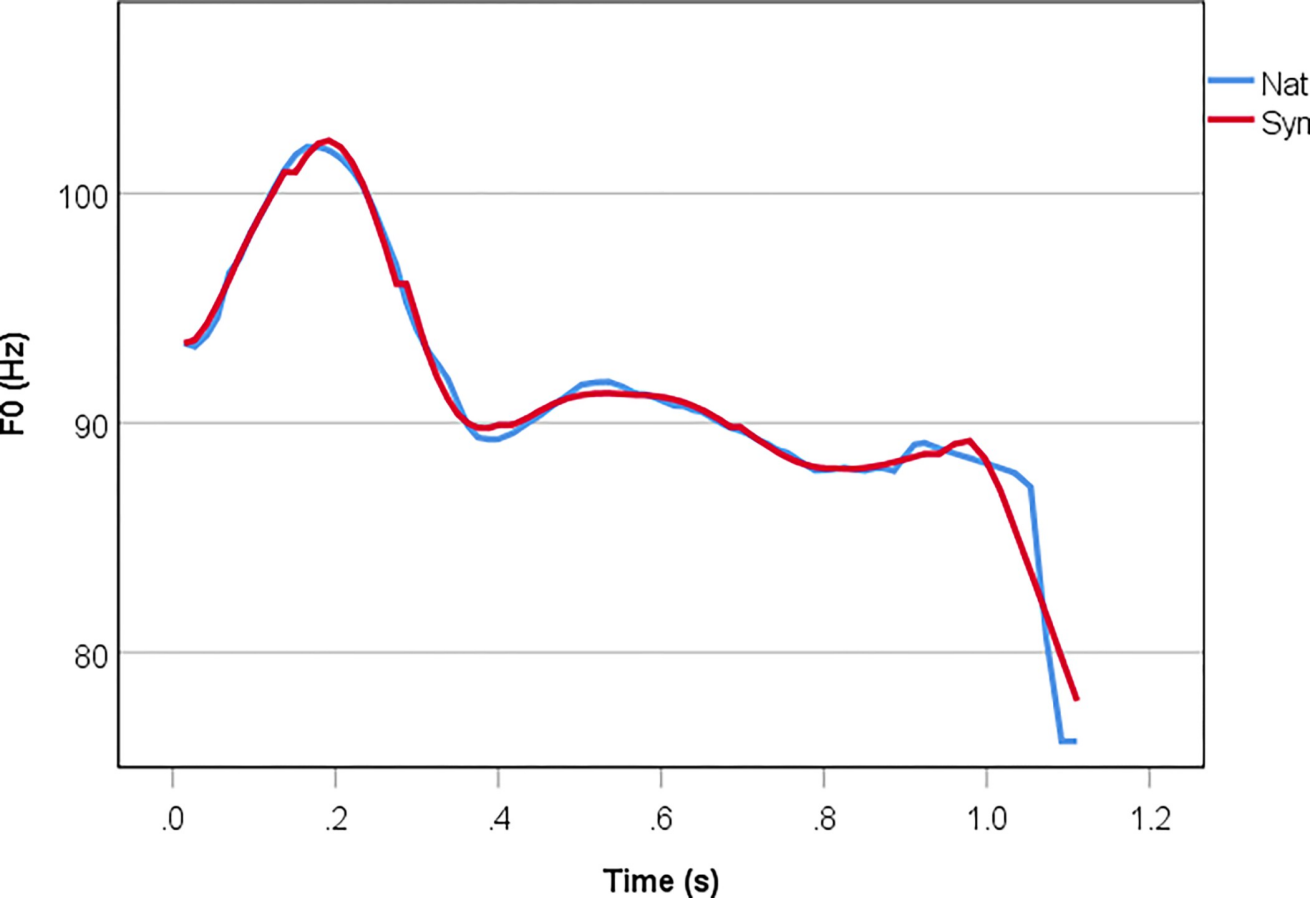
F0 contour of a natural vs. corresponding synthesised utterance (*me’i-ga mo’mo-o mi’ta*) in neutral focus. X-axis shows actual time.

To ensure that the resynthesised stimuli of different focus conditions differ only in F0, we had 16 base sentences on which to impose F0 contours generated by PENTAtrainer. These sentences consisted of the eight accent conditions × two lengths in Table 1. This means that for a given focus condition, non-F0 acoustic cues such as duration and intensity were held constant for all resynthesised stimuli. This is in contrast to [39] where each resynthesised stimulus was based on its respective natural utterance counterpart. Fig 3 illustrates the high synthesis accuracy of PENTAtrainer based on a neutral focus natural utterance vs. its synthesised counterpart (same base sentence in this case). The F0 contours closely overlapped each other, showing that in this example the synthesised utterance was highly similar to the natural one. Since some resynthesised stimuli do not share the same base sentence with their natural stimulus counterparts (to ensure minimal contrasts in F0), a direct assessment of synthesis accuracy like in [39] was not possible; instead, we justify the suitability of our resynthesised stimuli with a naturalness judgment task, as will be reported below (Experiment 2).

#### Participants

We recruited seven native listeners of Japanese (four male) for this pilot study. Their age range was 20 to 42 (*M* = 30.4, *SD* = 9.5). All were students who had moved to Hong Kong or England for less than six months at the time of the experiment. One participant had also lived in the USA for four years. None had reported any history of speech or hearing impairment. No participant in this task also took part in Experiment 2 which will be reported below. Written informed consent was obtained from all participants in this experiment and in Experiment 2. All experiments reported in this paper were approved by the UCL Research Ethics Committee (SHaPSetXU002).

#### Procedures

The experiment took place in a quiet room. Participants were seated in front of a laptop computer, which displayed the PRAAT ExperimentMFC interface (see S3 File). They wore circumaural headphones and listened to the stimuli consecutively. The entire experiment was conducted in Japanese. Participants were instructed to ‘determine which word was being emphasised’ with four options, namely ‘Word 1’, ‘Word 2’, ‘Word 3’, and ‘No emphasis’, which respectively corresponded to initial, medial (or penultimate), final, and neutral focus. They were also asked to respond as quickly as possible. There were 384 trials altogether (eight accent conditions × four focus conditions × two sentence types × three occurrences × two types of stimuli, i.e. natural vs. resynthesised). Each stimulus could be replayed up to three times.

### Result

The overall accuracy of focus identification was highly similar between natural (M = 49.1%, SD = 24.6%) and resynthesised (M = 47.6%, SD = 25.9%) stimuli. For final (natural = 42.3%, resynthesised = 45.8%) and neutral (natural = 50.3%, resynthesised = 63.4%) foci, resynthesised stimuli even appeared to yield better accuracy than natural stimuli, although the differences were not significant. A paired samples t-test showed that identification accuracy rates did not differ between the two types of stimuli, *t*(27) = 1.238, *p* = .227. These results thus indicate that focus cues in Japanese are mostly carried by F0, as the other cues that natural stimuli may contain did not provide clear advantages over F0. Given this finding, Experiment 2 will use resynthesised stimuli alone to explore the effects of pitch accent and focus location on focus perception in Japanese.

## Experiment 2

This experiment investigated the effects of focus location and accent condition on focus identification accuracy using only resynthesised stimuli. The research question is which word location under narrow focus is the most indistinguishable from neutral focus. We also asked the question of whether an UAU utterance with no narrow focus would yield the lowest identification accuracy since the lexical pitch accent may sound like medial narrow focus. We started by checking whether natural and resynthesised stimuli were equally natural-sounding to the participants, and then analyzed their focus identification accuracy using only resynthesised stimuli.

### Method

#### Participants

A new group of 16 native listeners of Japanese (3 male) were recruited in London for Experiment 2. All participants were born and raised in the Greater Tokyo area (Tokyo, Saitama, Kanagawa, and Chiba), and aged between 23 and 37 years (*M* = 27.9, *SD* = 4.0). Most participants had arrived in the UK for less than 12 months with the exception of one participant who had also spent two years in the USA prior to arriving in the UK. In subsequent analyses this listener was not found to behave differently from the other listeners in any discernable way. None reported any history of speech or hearing impairment.

#### Stimuli

A different subset of stimuli from the corpus described in Experiment 1 was used. The stimuli were produced by the same female speaker. These included both the short and long utterances in Table 1 but excluded interrogative stimuli for a more focused discussion on the interaction between the communicative functions of focus and pitch accent (see S1 Table in S2 File). In total, there were 128 tokens (eight accent conditions × four focus conditions × two types of stimuli × two lengths) from one female speaker (same as in Experiment 1) used in the naturalness judgment task and 64 resynthesised tokens (eight accent conditions × four focus conditions × two lengths) in the focus identification task. The generation of resynthesised stimuli followed the same procedure as in Experiment 1.

#### Procedures

The experiment took place in a quiet room. Participants were randomly assigned to one of two groups. One group judged long utterances in the naturalness rating task (eight accent conditions × four focus conditions × two types of stimuli = 64 trials) and short ones in the focus identification task (eight accent conditions × four focus conditions × three occurrences = 96 trials), and vice versa for the other group. Participants were seated in front of a laptop computer, which displayed the PRAAT ExperimentMFC interface, as in Experiment 1. They wore circumaural headphones and listened to the stimuli consecutively. In the naturalness rating task, participants rated each stimulus for naturalness on a 1 to 5 scale, with 5 being the most natural-sounding. In the focus identification task, participants performed focus identification with four options (Word 1 / 2 / 3 / No emphasis). Each stimulus could be replayed up to three times. After the naturalness rating task, participants could choose to take a break before beginning the focus identification task.

## Results

### Naturalness rating check

We were interested in whether Type of Stimuli (natural vs. resynthesised) affected how a listener rated the naturalness of stimuli (see S4 File). The grand mean rating of natural stimuli was 3.83 (*SD* = .641), which was close to that of resynthesised stimuli (*M* = 3.74, *SD* = .622). One-way repeated measures ANOVA showed that Type of Stimuli had no significant effect on naturalness judgment rating *F*(1,15) = .638, *p* = .437, suggesting that the two types of stimuli did not sound different to native listeners in terms of naturalness. Based on the similarity of naturalness between model-generated and natural stimuli as found here as well as in Experiment 1, we now proceed to the following analyses using only resynthesised stimuli.

### Focus identification

**Fig 4:**
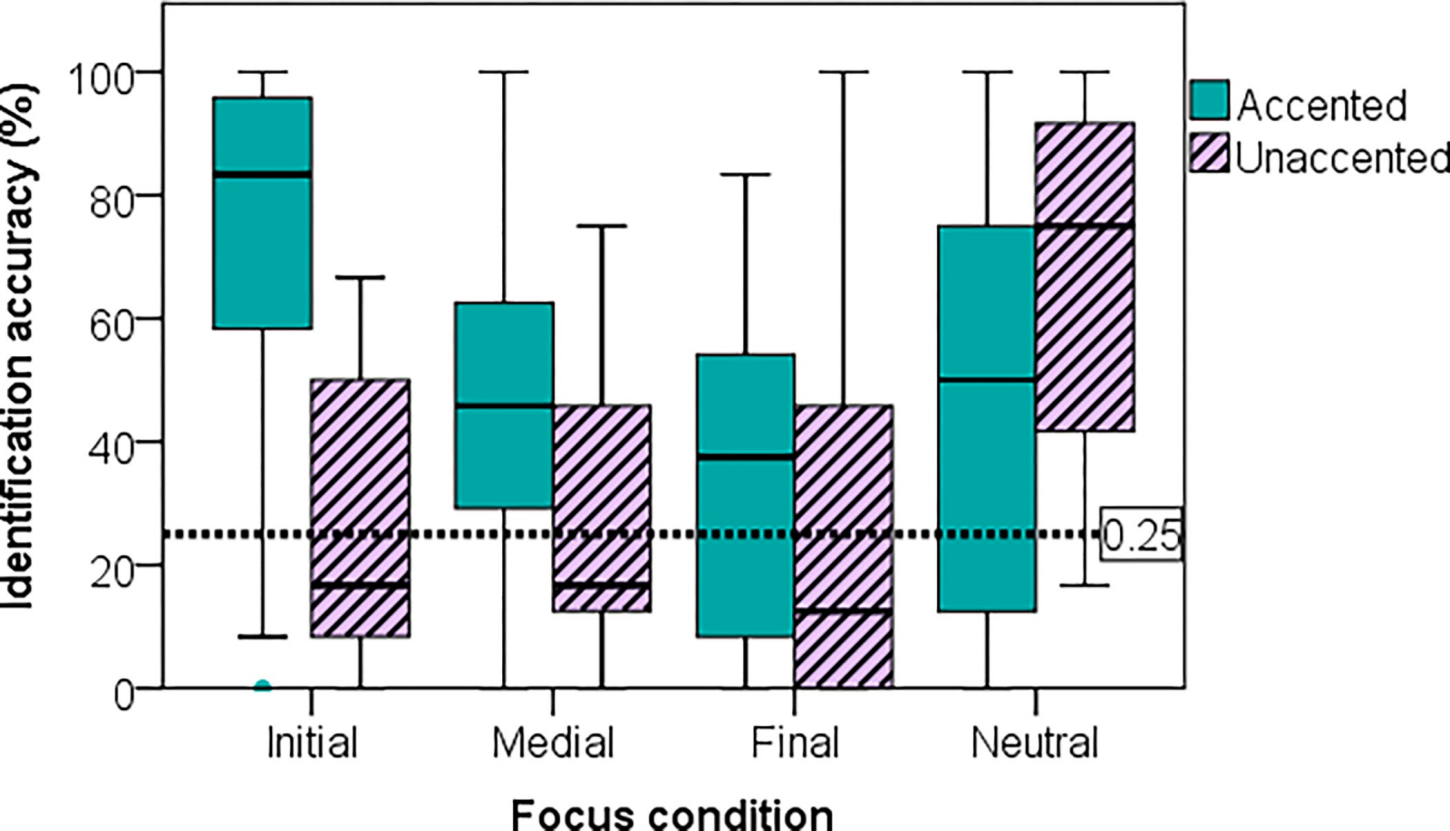
Mean identification accuracy by focus condition and accent condition of the focused word (or of Word 1 in case of neutral focus).

Fig 4 shows that, in general, focus was identified more accurately when the word bearing narrow focus was lexically accented (see S5 File). For all narrow focus conditions, an accented focus (solid box) yielded higher identification accuracy than an unaccented focus (striped box). For a neutral focus utterance (in this figure), the accent condition was that of Word 1, and in this case pitch accent on the first word appeared to make it hard for the sentence to be perceived as neutral focus (47% vs. 67%, chance = 25%, i.e., dotted line in Fig 4). On the whole, the most easily identified focus condition in statements was neutral (*M* = 57%, *SD* = 31%), followed by initial (M = 49%, SD = 22%), medial (*M* = 39%, *SD* = 20%), and final (*M* = 31%, *SD* = 28%). Refer to S2 Table in S2 File for corresponding reaction time data.

The combination of lexical accent conditions also affected identification accuracy. Table 2 shows that identification accuracy was the lowest in UAU (*M* = 32%, *SD* = 15%), whereas focus in all-accented (AAA) utterances was the most correctly identified (*M* = 56%, *SD* = 18%).

**Table 2:** Mean identification accuracy (chance = 25%) by accent combination and focus location. Accent combinations are in descending order of mean identification accuracy. Cell colouring signals the percentage of correct responses (25% < green < 50% < yellow < 75% < red) (colour online).

	Accuracy (%)					
Accent comb.	Initial	Medial	Final	Neutral	*M*	*SD*
**AAA**	79	63	38	46	56	18
**AUA**	77	29	44	44	48	20
**UAA**	27	52	50	60	47	14
**UUU**	33	35	38	75	45	20
**AAU**	63	48	27	42	45	15
**AUU**	69	21	17	58	41	26
**UUA**	21	38	10	77	36	29
**UAU**	23	27	25	54	32	15

A logistic mixed-effects model was fitted to the identification accuracy data using the R package *lmer4* [42 version 1.1–15]. The final model in Table 3 (left) was built by adding significant fixed effects one by one on a simpler model. The significance of each fixed effect was determined using the *anova()* function, by comparing a model with the fixed effect in question and a model without. The final model contained the fixed effects of focus location (‘Focus’ in Table 3, χ^2^(3) = 66.151, *p* < .001), accent combination (‘Accent’, χ^2^(7) = 32.058, *p* < .001), lexical accent condition of the focused word (‘FocusAccent’ henceforth, χ^2^(1) = 19.034, *p* < .001), and the interaction between Focus and FocusAccent (χ^2^(3) = 112.071, *p* < .001). ‘FocusAccent’ was included to examine the possible effect of lexical prosody on focus identification (see §1.2).

**Table 3:** Model summary: Correct response ~ Focus + Accent + FocusAccent + Focus × FocusAccent + (Focus | Group:Subject). *Pr(>|z|)*-values: * < .05, ** < .01, *** < .001. In the parameters, ‘I’ = Initial focus, ‘M’ = Medial, ‘F’ = Final, ‘N’ = Neutral, ‘A’ = Accented, ‘U’ = Unaccented.

Parameters	Fixed				Random
	*β*	*SE*	*z*		Participant
					SD
(Intercept)	-.072	.555	-.129		1.977
Focus (Final)	-1.893	.683	-2.771	**	2.548
Focus (Initial)	-.752	.665	-1.131		2.515
Focus (Medial)	-1.122	.647	-1.735	.	2.455
Accent (AAA)	1.044	.292	3.579	***	
Accent (AAU)	.410	.285	1.437		
Accent (AUA)	.884	.306	2.888	**	
Accent (AUU)	.485	.303	1.600		
Accent (UAA)	.874	.265	3.303	***	
Accent (UUA)	.514	.283	1.819	.	
Accent (UUU)	1.134	.273	4.152	***	
FocusAccent	-1.358	.325	-4.176	***	
Focus (F) x FocusAccent	1.674	.446	3.755	***	
Focus (I) x FocusAccent	3.896	.399	9.760	***	
Focus (M) x FocusAccent	2.351	.429	5.485	***	

For all the fixed factors, contrasts were recoded: for focus location, we used dummy coding with Neutral as the reference level; the same was done to accent combination, with UAU as reference; the accent condition of the focused word (‘FocusAccent’ in Table 3) was deviation-coded. Random effects included intercepts for subjects nested within groups (hearing long vs. short stimuli), and by-subject random slopes for Focus. Adding more factors led to the model failing to converge.

Table 3 (left) shows the summary of this model. With regard to focus location, neutral focus was significantly more accurately identified than final focus (*β* = -1.893, SE = .683, z = -2.771). For accent combination, condition UAU was significantly less correctly identified than AAA, AUA, UAA, and UUU (all *Pr(>|z|)* < .01). Finally, although on the whole lexically accented narrow foci (51% correct) were more accurately identified than otherwise (38% correct), Fig 4 shows that the opposite was true for neutral focus. The three significant (all *Pr(>|z|)* < .001) interaction terms (Focus x FocusAccent) show that compared with neutral focus, a lexically accented narrow focus was associated with significantly more correct responses than an unaccented narrow focus. See also Table 4 for participants’ mean response rates under each target condition.

**Table 4:** Confusion matrix summarizing mean response rates in Experiment 2.

		Response			
		Initial	Medial	Final	Neutral
Target	Initial	**49%**	10%	4%	38%
	Medial	15%	**39%**	5%	41%
	Final	13%	17%	**31%**	39%
	Neutral	18%	20%	5%	**57%**

## Discussion

### Summary of findings

This study set out to answer three questions: (i) how Japanese listeners perform in focus identification in general when F0 is the only cue available, (ii) which focus location is more accurately identified than others, and (iii) if lexical pitch accent affects focus perception.

Firstly, in terms of overall identification accuracy, participants seemed to perform relatively poorly across all focus locations. The accuracy rates ranged from 31% for final focus (among which the UUA condition was as low as 10%, chance = 25%) to 57% for neutral focus (among which the UUA condition was 77%). These figures might lead one to question the reliability of the resynthesised stimuli, especially when no natural stimuli were used in Experiment 2 for comparison. However, for sentence-initial narrow accented focus, the mean correct identification rate was as high as > 70% (Fig 4). In fact, these figures are comparable with [13], in which focus identification accuracy ranged from 23% to 77%. Taken together with the results of Experiment 1 that the resynthesised stimuli did not significantly differ from their natural counterparts, the overall low identification accuracy can instead be attributed to the influences of specific accent and focus locations, as will be discussed below.

### Why is focus perception so hard in Japanese?

The goal of this study is to find out why focus perception in Japanese is not as robust as in some other languages, such as Mandarin [16, 43], English [44], and Hindi [45]. Those languages share with Japanese an important property of focus prosody, namely, post-focus compression of pitch range (PFC) that has been demonstrated to be beneficial for focus perception in these languages [39, 40] as compared to non-PFC languages such as Cantonese [46] and Southern Min [47]. The low focus identification rate in Japanese despite the presence of PFC is a puzzle that the present study intends to solve. We have attempted to achieve this by examining two likely factors that may play a major role in focus perception, namely, focus location and accent condition.

For this purpose, we used a method that can manipulate focus relevant F0 cues while holding constant other potential cues, such as duration and intensity. The manipulations performed were different from conventional approaches, as they were done using a computational modeling tool—PENTAtrainer [4] to make sure the manipulated intonation closely resembles that of natural speech. The effectiveness of the method was confirmed in Experiment 1 which showed that the resynthesised and natural stimuli were perceptually similar to natural utterances in both focus identification rate and naturalness. This demonstrated that the F0 manipulation by PENTAtrainer regenerated sufficient focus cues despite the absence of non-F0 cues such as duration, intensity, and voice quality.

Experiment 2 therefore investigated the effects of focus location and accent condition on focus identification using only resynthesised stimuli. In terms of accent type, in general, focus identification was more accurate when the focused words were accented than when they were unaccented. In terms of focus location, initial focus was the most accurately identified, followed by medial focus and final focus. Upon closer examination, the effects of both accent type and focus location are closely related to how they interfered with the effective realisation of PFC in the language. Only in accented words was there not only on-focus pitch range expansion, but also PFC. In unaccented words, in contrast, although there is some on-focus pitch range expansion, PFC of pitch range was absent, as can be seen in Fig 1. This suggests that the lack of PFC in unaccented words is a major source of difficulty in focus identification for our listeners.

In terms of focus location, a general trend was that the earlier the focus, the higher the identification rate. Of particular interest is the low identification rate for medial focus (39%) as compared to initial focus (49%). Due to the short sentences used in this study, sentence medial focus is also penultimate focus. As found in a number of studies, penultimate focus is not effectively marked if a language SOV, which is the case with Japanese. This is because the default prosody in a SOV sentence already somewhat resembles PFC prosody, because verbs tend to have lower F0 than nouns, which makes a penultimate focus not far different from a neutral focused SOV sentence [6, 7, 27]. Meanwhile, the low identification rate of final focus (31%) is in line with the well-established observation that final focus is not highly distinguishable from neutral focus [29].

With regard to accent combination, our speculation that the UAU condition would yield the lowest focus identification accuracy was confirmed. The AAA condition yielded the highest identification accuracy (56%) while the worst was UAU (32%). Besides, AAU and AUU also yielded significantly lower focus identification accuracy than AAA. As discussed earlier, the UAU condition was expected to be the most confusable because under neutral focus the sentence-medial pitch accent could be misperceived as a narrow focus, thus leading to misidentification. For AAU, listeners only correctly identified final focus 27% of the time (see Table 2), compared with AAA (38% for final focus), echoing the significant main effect of FocusAccent. The same is true for the AUU condition, where medial focus was correctly identified only 21% of the time (cf. 63% for AAA) and final focus only 17% of the time (cf. 38% for AAA), further confirming that narrow focus on an unaccented word is hard to identify for native listeners. Taken together, we argue that the effects of (i) accent condition of the focused word and (ii) combination of accent conditions within a sentence both point to the lack of reliable realisation of PFC as the likely source of difficulty of focus encoding in Japanese.

### Model-generated resynthesised stimuli for speech perception studies

The generation of perception stimuli in the present study differs from the conventional approaches whereby various aspects of F0 contours, such as height, slope, location of turning point, etc., are directly manipulated. PENTAtrainer [4] used in this study generates intonational contours that mimic natural F0 contours in two ways. First, target approximation (TA) in the PENTA model mimics the natural articulation process of pitch production [40], guaranteeing that the generated local F0 trajectories are largely free of artefacts such as F0 slopes that are too sharp or shallow and especially unnatural alignments with the syllable. Second, the function-specific F0 properties are learned directly, in the modeling process, from multiple tokens of natural speech. This has allowed us to manipulate F0 contours appropriate for pitch accents and focus condition in a highly natural way. These contours were then imposed onto an originally neutral-focus natural utterance to generate stimuli that are free of the contributions of non-F0 cues. The naturalness and effectiveness of the model generated stimuli were confirmed by the results from both Experiment 1 and Experiment 2.

### Caveats and limitations

Several caveats need to be taken into account when interpreting the present results. Firstly, the focus locations in this study are different in terms of segmental content and length. This is because when designing the stimuli we prioritised the need of maximising sonorant sounds in the stimuli and the need of ensuring minimal pairs contrasting in lexical pitch accent, which were rare in the first place. As a result, we were unable to make target words in the three word locations more comparable than they are. Future studies could verify the current findings using techniques such as reiterant speech [see e.g., 48 who used reiterant speech to study lexical accent in Japanese]. Secondly, as our design incorporated several fixed factors at the same time, to avoid excessively long testing sessions, we only had two base sentences (long and short); future research could revisit a subset of these factors and use more base sentences to test our conclusions. Thirdly, we achieved strict experimental control in our stimuli at the expense of semantic naturalness in some of the sentences. On this point, however, we would like to note that the semantic unnaturalness could have contributed to reduced or exaggerated overall focus identification accuracy, but none of the variable patterns related to accent type or focus location seems to be attributable to the lack of semantic naturalness. That said, it would be desirable for future studies to find a better balance between semantic naturalness and experimental control.

## Conclusion

This study set out to investigate why the perception of prosodic focus in Japanese is not as robust as in some other language that share an important focus marking cue, namely PFC. The results from the experiments revealed that the realisation of PFC was heavily interfered with by two characteristics of Japanese, namely lexical pitch accent and word order. The blocking PFC by lexically unaccented words as found in previous research was shown in the present study to also effectively impede the perception of focus. The SOV word order of Japanese was also found to make focus perception difficult given that its associated global F0 contours partly resembles those of PFC. These findings were made in the present study by the use of PENTAtrainer, a computational modeling tool for speech prosody, which demonstrated a clear potential for modeling-based perception research.

## Supporting information

S1 File (PDF)

S2 File (PDF)

S3 File (PRAAT)

S4 File (CSV)

S5 File (CSV)
