# Supplementary material for: Focus perception in Japanese: Effects of lexical accent and focus location
Source: PLoS One. 2022 Sep 22;17(9):e0274176. doi: 10.1371/journal.pone.0274176 (PMC9499294; doi:10.1371/journal.pone.0274176)
Supplement: S1 File — (PDF) [file pone.0274176.s001.pdf]

Supplemental material 1

Full list of stimuli used in the present study. Pitch accent is marked with a "’" after the accented mora.

1. ***Me’i-ga momo-o mi’ta."*** "May saw peach."
2. ***Me’i-ga mo’mo-o mi’ta."*** "May saw thigh."
3. ***Me’i-ga momo-ni nita."*** "May resembled peach."
4. ***Me’i-ga mo’mo-ni nita."*** "May resembled thigh."
5. ***Mei-ga momo-o mi’ta."*** "Niece saw peach."
6. ***Mei-ga mo’mo-o mi’ta."*** "Niece saw thigh."
7. ***Mei-ga momo-ni nita."*** "Niece resembled peach."
8. ***Mei-ga mo’mo-ni nita."*** "Niece resembled thigh."
9. ***Mu’umin-ga bu’dou-o mi’ta."*** "Moomin saw martial arts."
10. ***Mu’umin-ga budou-o mi’ta."*** "Moomin saw grapes."
11. ***Mu’umin-ga bu’dou-ni nita."*** "Moomin resembled martial arts."
12. ***Mu’umin-ga budou-ni nita."*** "Moomin resembled grapes."
13. ***Noumin-ga bu’dou-o mi’ta."*** "Farmer saw martial arts."
14. ***Noumin-ga budou-o mi’ta."*** "Farmer saw grapes."
15. ***Noumin-ga bu’dou-ni nita."*** "Farmer resembled martial arts."
16. ***Noumin-ga budou-ni nita."*** "Farmer resembled grapes."
