# Supplementary material for: Focus perception in Japanese: Effects of lexical accent and focus location
Source: PLoS One. 2022 Sep 22;17(9):e0274176. doi: 10.1371/journal.pone.0274176 (PMC9499294; doi:10.1371/journal.pone.0274176)
Supplement: S2 File — (PDF) [file pone.0274176.s002.pdf]

Supplemental material 2

Mean reaction times in Experiment 2 by accent combination.

	RT (s)	
**Accent comb.**	***M***	***SD***
**UAA**	2.83	1.09
**AAU**	2.84	1.13
**AAA**	2.88	1.27
**AUA**	2.89	1.26
**UAU**	2.89	1.32
**AUU**	2.92	1.31
**UUA**	3	1.63
**UUU**	3.09	1.33

Confusion matrix comparing focus identificaiton accuracy by accent condition x focus location

	Target	Initial	Medial	Final	Neutral
	Response				
	Initial	**79.17%**	18.75%	8.33%	14.58%
AAA	Medial	2.08%	**62.50%**	25.00%	33.33%
	Final	4.17%	6.25%	**37.50%**	6.25%
	Neutral	14.58%	12.50%	29.17%	**45.83%**
	Response				
	Initial	**62.50%**	14.58%	22.92%	25.00%
AAU	Medial	6.25%	**47.92%**	10.42%	31.25%
	Final	6.25%	4.17%	**27.08%**	2.08%
	Neutral	25.00%	33.33%	39.58%	**41.67%**
	Response				
	Initial	**77.08%**	22.92%	22.92%	37.50%
AUA	Medial	4.17%	**29.17%**	10.42%	14.58%
	Final	2.08%	6.25%	**43.75%**	4.17%
	Neutral	16.67%	41.67%	22.92%	**43.75%**
	Response				
	Initial	**68.75%**	29.17%	20.83%	27.08%
AUU	Medial	6.25%	**20.83%**	16.67%	10.42%
	Final	4.17%	4.17%	**16.67%**	4.17%
	Neutral	20.83%	45.83%	45.83%	**58.33%**
	Response				
	Initial	**27.08%**	10.42%	6.25%	10.42%
UAA	Medial	22.92%	**52.08%**	18.75%	22.92%
	Final	0.00%	6.25%	**50.00%**	6.25%
	Neutral	50.00%	31.25%	25.00%	**60.42%**
	Response				
	Initial	**22.92%**	10.42%	6.25%	14.58%
UAU	Medial	14.58%	**27.08%**	20.83%	25.00%
	Final	4.17%	8.33%	**25.00%**	6.25%
	Neutral	58.33%	54.17%	47.92%	**54.17%**
	Response				
	Initial	**20.83%**	4.17%	8.33%	12.50%
UUA	Medial	8.33%	**37.50%**	25.00%	6.25%
	Final	4.17%	2.08%	**10.42%**	4.17%
	Neutral	66.67%	56.25%	56.25%	**77.08%**
	Response				
	Initial	**33.33%**	8.33%	8.33%	6.25%
UUU	Medial	12.50%	**35.42%**	8.33%	12.50%
	Final	4.17%	2.08%	**37.50%**	6.25%
	Neutral	50.00%	54.17%	45.83%	**75.00%**

Log-gamma mixed-effects analysis of reaction time data (N = 676) in Experiment 2. RTs of incorrect responses have been excluded from this model. Planned contrasts for both Focus and Accent have been recoded so that each level was compared with the adjacent one in terms of overall RT. In the parameters, 'I' = Initial, 'M' = Medial, 'F' = Final, 'N' = Neutral, 'A' = Accented, 'U' = Unaccented.

Model summary: Reaction times ~ Focus + Accent + (1 | Group:Subject). *Pr(>|z|)*-values: * < .05, ** < .01, *** < .001.

Parameters	Fixed				Random
					Participant
	β	*SE*	*t*		*SD*
(Intercept)	**1.000**	**.043**	**23.194**	***	.099
Focus (I - M)	**-.159**	**.043**	**-3.720**	***	
Focus (M - N)	**-.154**	**.052**	**-2.933**	**	
Focus (N - F)	**-.205**	**.051**	**-4.036**	***	
Accent (UAA - AAU)	-.060	.062	-.956		
Accent (AAU - AAA)	**-.193**	**.083**	**-2.328**	*	
Accent (AAA - AUA)	**-.356**	**.094**	**-3.802**	***	
Accent (AUA - UAU)	**-.447**	**.100**	**-4.453**	***	
Accent (UAU - AUU)	**-.444**	**.097**	**-4.576**	***	
Accent (AUU - UUA)	**-.348**	**.088**	**-3.967**	***	
Accent (UUA - UUU)	**-.321**	**.063**	**-5.077**	***	

Confusion matrix summarizing reaction time data corresponding to Table 4 (Experiment 2).

		RT (s)			
		Initial	Medial	Final	Neutral
Target	Initial	**2.49**	3.33	2.65	2.91
	Medial	2.70	**2.70**	3.46	3.02
	Final	2.66	3.08	**2.92**	3.10
	Neutral	2.50	2.79	2.91	**2.77**

Confusion matrix summarizing mean reaction times in Experiment 3 (colour online). The first row shows the lexical accent condition (initial accent vs. unaccented) of the focused word, or the first word in the neutral focus condition. The second column represents the location of the word replaced by pink noise ('Pre': pre-focus 'On': on-focus; 'Post': post-focus; 'Far pre': word before pre-focus; 'Far post': word after post-focus). The left column shows narrow focus conditions (I = initial, M = medial, F = final). Cell colouring indicates the smallest (green) and largest (red) four values.

	Accented						Unaccented						Combined					
	Far pre	Pre	On	Post	Far post	N	Far pre	Pre	On	Post	Far post	N	Far pre	Pre	On	Post	Far post	N
I			2.57	2.45	2.46				2.64	2.71	2.73				2.61	2.58	2.60	
M		2.78	2.58	2.61		2.53		2.79	2.57	2.77		2.82		2.79	2.58	2.69		2.67
F	2.75	2.68	2.81				2.87	3.02	2.57				2.81	2.85	2.69			
Total	2.75	2.73	2.66	2.53	2.46		2.87	2.91	2.59	2.74	2.73		2.81	2.82	2.62	2.63	2.60	

Log-gamma mixed-effects analysis of reaction time data (N = 534) in Experiment 2. RTs of incorrect responses have been excluded from this model. Planned contrasts for Mask have been recoded so that each level was compared with the adjacent one in terms of overall RT.

Model summary: Reaction times ~ (1 | Group:subject) + Mask + FocusAccent. *Pr(>|z|)*-values: * < .05, ** < .01, *** < .001. Mask refers to the word replaced by pink noise (levels as in Table 5).

Parameters	Fixed				Random
					Participant
	β	*SE*	*t*		*SD*
(Intercept)	**.985**	**.053**	**18.515**	***	.110
Mask (Far post - On)	**-.228**	**.069**	**-3.324**	***	
Mask (On - Post)	-.077	.104	-.743		
Mask (Post - N)	**-.232**	**.097**	**-2.384**	*	
Mask (N – Far pre)	**-.381**	**.092**	**-4.142**	***	
Mask (Far pre - Pre)	**-.188**	**.063**	**-2.983**	**	
FocusAccent	**-.074**	**.026**	**-2.881**	**	
